# High ligation of the inferior mesenteric artery during sigmoid colon and rectal cancer surgery increases the risk of anastomotic leakage: a meta-analysis

**DOI:** 10.1186/s12957-018-1458-7

**Published:** 2018-08-02

**Authors:** Jinshui Zeng, Guoqiang Su

**Affiliations:** Department III of Gastrointestinal Surgery, First Affiliated Hospital of Xiamen University , Xiamen, 361003 Fujian China

**Keywords:** Inferior mesenteric artery, High ligation, Low ligation, Sigmoid colon cancer, Rectal cancer, Anastomotic leakage

## Abstract

**Background:**

The ideal level of ligation of the inferior mesenteric artery (IMA) during curative resection of sigmoid colon and rectal cancer is still controversial. The aim of this meta-analysis was to examine the impact of high ligation and low ligation of the IMA on anastomotic leakage, overall morbidity, postoperative mortality, and oncological outcomes in patients undergoing surgery for sigmoid colon and rectal cancer.

**Methods:**

PubMed, EMBASE, Web of Science, and BioMed Central databases were searched to identify relevant articles published from May 1953 to March 2018. A total of 18 articles (14 non-randomized studies and 4 randomized clinical trials) were identified. Review Manager 5.3 software was used for analysis of data. The pooled odds ratio (OR) and weighted mean difference (WMD), with 95% CI, were calculated using either the fixed effects model or random effects model.

**Results:**

Of the 5917 patients included in this meta-analysis, 3652 patients underwent low ligation of the IMA and 2265 patients underwent high ligation of the IMA. Anastomotic leakage rate was 9.8% in high ligation patients vs. 7.0% in low ligation patients; the risk of anastomotic leakage was significantly higher in high ligation patients (OR = 1.33; 95% CI 1.10–1.62; *P* = 0.004). What is more, overall morbidity was also significantly higher in high ligation patients (OR = 1.39; 95% CI, 1.05–1.68; *P* = 0.05). Postoperative mortality, number of harvested lymph nodes, overall recurrence rate, and 5-year survival rate did not differ significantly between the two groups.

**Conclusion:**

Low ligation of the IMA during curative resection of sigmoid colon and rectal cancer appears to be associated with lower risk of anastomotic leakage and overall morbidity. However, there was no significant advantage of low ligation over high ligation of IMA in terms of postoperative mortality, the number of harvested lymph nodes, overall recurrence rate, or 5-year survival rate.

## Background

Colorectal cancer (CRC) is the third most common cancer in the world and the fourth most common cause of cancer-related death [1]. The standard operative procedure for curative resection of rectal cancer and sigmoid colon cancer includes removal of the tumor, wide resection of the colonic mesentery, and ligation of inferior mesenteric vessels [2]. There are two options for ligation of the inferior mesenteric artery (IMA): (1) high ligation, with ligation of the left colic artery (LCA), and (2) low ligation, with preservation of the LCA [3]. Ever since 1908, when Miles et al. recommended low ligation of the IMA [4] and Moynihan et al. recommended high ligation of the IMA [5], the controversy over the level of ligation of the IMA has persisted.

Lymph node metastasis is a crucial factor affecting the outcome of sigmoid colon and rectal cancer surgery [6], and ligation of the IMA root can facilitate removal of the surrounding lymph nodes during curative resection of sigmoid colon and rectal cancer. Thus, the high ligation of the IMA can improve lymph node retrieval rates and enable accurate tumor staging [7]. However, it is uncertain whether there are long-term survival benefits to be had with high ligation [8–10]. Seike et al. reported significant reduction in blood flow to the anastomosis after high ligation of the IMA [11]. Others have argued that high ligation is associated with higher risk of hypogastric plexus injury, whereas low ligation avoids damage to the pre-aortic nerves and preserves the blood supply to the anastomosis [12, 13]. Meanwhile, several clinical studies have demonstrated that high ligation allows complete mobilization of the proximal colonic limb and thus helps reduce anastomotic tension [14]. Interestingly, previous meta-analyses have arrived at different conclusions about the incidence of anastomotic leakage (AL) with the two approaches. Fan et al. reported that low ligation of the IMA may lower the risk of AL, whereas pooled data from the studies of Cirocchi et al. and Yang et al. showed that there was no significant difference in the incidence of AL with the two approaches [5, 8, 9].

This meta-analysis was performed to compare the effect of high versus low ligation of the IMA during curative resection of sigmoid colon and rectal cancer on outcomes such as AL, overall morbidity, postoperative mortality, recurrence, and survival.

## Methods

### Study selection

This meta-analysis was conducted in March 2018. We searched the databases of PubMed, EMBASE, Web of Science, and BioMed Central for all articles referring to high and low ligation of the IMA during curative resection of sigmoid colon and rectal cancer. The following MeSH terms were used for the search: “inferior mesenteric artery,” “left colic artery,” “colorectal cancer,” “high ligation,” “low ligation,” and “anastomotic leakage.” Two authors independently reviewed and assessed the titles and/or abstracts of the studies and excluded obviously irrelevant articles. The full texts of the remaining studies were examined to decide whether they met the inclusion criteria. In addition, we manually reviewed the reference lists of potentially relevant articles to broaden the search.

### Inclusion criteria

Studies were eligible if they were (1) non-randomized studies or randomized controlled trials (RCTs) comparing high ligation vs. low ligation of the IMA during curative resection of sigmoid colon and rectal cancer, regardless of the surgical approach (open or laparoscopic) and surgical procedure (elective or emergency). Articles in all languages were eligible for inclusion. In cases of duplicate articles, only the latest published version was included.

### Exclusion criteria

Studies were excluded if (1) the outcome of the primary end point (AL) was not reported; (2) there was no control group; and (3) the full text could not be accessed. Review articles, letters, case reports, and meta-analyses were not considered.

### End points

The primary end point was the AL rate. The secondary end points were overall morbidity, postoperative mortality, number of harvested lymph nodes, overall recurrence rate, and 5-year survival rate.

### Data items

The following data were extracted from the included studies: the number of patients in each treatment group, year and country of the study, type of study (non-randomized study vs. RCT), type of surgery (high ligation vs. low ligation), study inclusion and exclusion criteria, and the outcomes measured. Relevant data were extracted by two authors independently, and any disagreement was resolved by discussion.

### Assessment of the quality

The quality and bias risk of the included studies was assessed independently by two authors; disagreements were settled by discussion. The Newcastle–Ottawa Scale (NOS) [15] was used to assess the quality of non-randomized clinical studies. Studies were judged on the basis of criteria such as the quality of patient selection, ascertainment of exposure, comparability of groups, and outcomes of patients. The total NOS score ranges from 0 to 9 stars; a score of ≥ 6 stars indicates high quality. The Jadad scoring system [16] was used to assess the bias risk of the RCTs. This scoring system is based on three specific items: randomization, blinding, and withdrawals and dropouts. The total score ranges from 0 to 5; a score of ≤ 2 indicates poor-quality evidence and a score of ≥ 3 indicates high-quality evidence.

### Statistical analysis

Statistical analysis was performed using the statistical software Review Manager Version 5.3 (Copenhagen: The Nordic Cochrane Centre, the Cochrane Collaboration, 2014). The odds ratios (OR) and the weighted mean differences (WMD), with 95% confidence intervals (CIs), were calculated for dichotomous and continuous variables, respectively. Homogeneity among the studies was assessed using the chi-square test and the inconsistency statistics (*I*^2^). Statistically significant homogeneity was indicated by a chi-square test with *P* < 0.10 [17]. In addition, *I*^2^ > 25%, *I*^2^ > 50%, and *I*^2^ > 75% indicated low, moderate, and high heterogeneity, respectively [18]. When there was no heterogeneity among the studies, the Mantel-Haenszel method was adopted to be combined with the ORs or the inverse variance method was used to be combined with the WMDs for a fixed effects model [19, 20]. If there was heterogeneity, the random effects model was adopted for the pooled analysis (DerSimonian and Laird method) [21]. The *Z* test (and the related *P* value) was used to assess overall effect. Statistical significance was at *P* ≤ 0.05. Sensitivity analysis was performed to explore statistical heterogeneity. Publication bias was assessed with funnel plots.

## Results

### Description of the studies

**Fig. 1:**
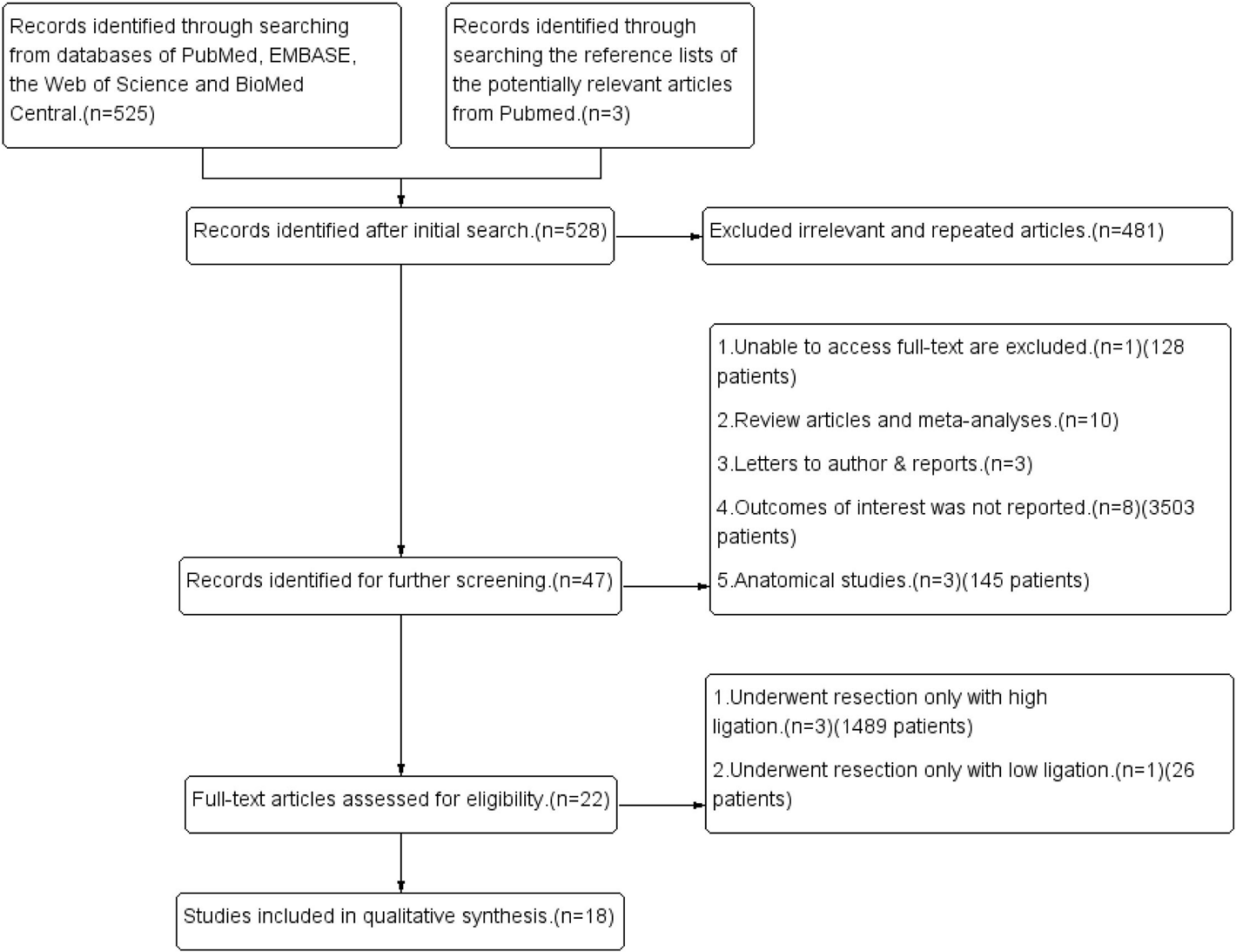
Flow diagram showing the process of literature search

Figure 1 shows the process of study selection for this meta-analysis. A total of 18 articles [22–39] published between July 1992 and March 2018 were included for this meta-analysis (Table 1). They included 14 non-randomized studies [22–29, 31, 33–36, 38] and 4 RCTs [30, 32, 37, 39]. The total number of patients was 5917, which included 3652 patients who underwent low ligation of the IMA and 2265 patients who underwent high ligation.

**Table 1:** Characteristics of the studies included in this meta-analysis

Study	Country	Year	Diagnosis	Type of surgery	Surgical treatment of IMA (no. of patients)		Type of study
					High ligation	Low ligation	
Corder AP	UK	1992	Rectal cancer	TME	91	52	Retrospective cohort
Hall NR	UK	1995	Left-side colorectal cancer	Curative resection	30	32	Retrospective cohort
Komen N	Netherlands	2011	Rectal cancer	TME	16	17	Retrospective cohort
Sekimoto M	Japan	2011	Sigmoid and rectal cancer	Laparoscopic resection	27	45	Retrospective cohort
Rutegard M	Sweden	2012	Rectal cancer	AR	818	1101	Retrospective cohort
Hinoi T	Japan	2013	Rectal cancer	Laparoscopic resection	304	584	Retrospective cohort
Yamamoto M	Japan	2014	Sigmoid and rectal cancer	Laparoscopic resection	91	120	Retrospective cohort
Bostrm P	Sweden	2015	Rectal cancer	AR	334	388	Retrospective cohort
Matsuda K	Japan	2015	Rectal cancer	AR	51	49	RCT
Huang J	China	2016	Rectal cancer	Laparoscopic resection	87	29	Retrospective cohort
Niu JW	China	2016	Rectal cancer	Laparoscopic AR	45	52	RCT
Rutegard M	Japan	2016	Rectal cancer	AR	5	18	Retrospective cohort
Yasuda K	Japan	2016	Colorectal cancer	Curative resection	42	147	Retrospective cohort
Zhang LY	China	2016	Rectal cancer	Laparoscopic resection	42	61	Retrospective cohort
Zhang YD	China	2016	Rectal cancer	Laparoscopic LAR	84	132	Retrospective cohort
Guo YC	China	2017	Rectal cancer	Radical resection	29	28	RCT
Mihara Y	Japan	2017	Colorectal cancer	NA	117	745	Retrospective cohort
Zhou JM	China	2018	Rectal cancer	Laparoscopic resection	52	52	RCT

*TME* total mesorectal excision, *AR* anterior resection, *LAR* low anterior resection, *NA* not available, *RCT* randomized clinical trial

Table 2 shows the results of quality assessment of the non-randomized studies with NOS. Table 3 shows the results of bias risk assessment of the RCTs with the Jadad scoring system. Table 4 lists the outcomes of interest reported in patients receiving high and low ligation of the IMA.

**Table 2:** Quality of non-randomized studies as assessed by the Newcastle–Ottawa Scale

Study	Year	Selection				Comparability	Outcome			Score
		1	2	3	4	1	1	2	3	(Max 9)
		(Max 1)	(Max 1)	(Max 1)	(Max 1)	(Max 2)	(Max 1)	(Max 1)	(Max 1)	
Corder AP	1992	*	*	*	*	*	*	–	*	7
Hall NR	1995	*	*	*	*	**	*	–	–	7
Komen N	2011	*	*	*	*	*	*	*	*	8
Sekimoto M	2011	*	*	*	*	*	*	–	*	7
Rutegard M	2012	*	*	*	*	*	*	*	*	8
Hinoi T	2013	*	*	*	*	**	*	*	–	8
Yamamoto M	2014	*	*	*	*	*	*	*	*	8
Bostrm P	2015	*	*	*	*	*	*	*	*	8
Huang J	2016	*	*	*	*	**	*	*	*	9
Rutegard M	2016	*	*	*	*	*	*	*	*	8
Yasuda K	2016	*	*	*	*	*	*	*	*	8
Zhang LY	2016	*	*	*	*	**	*	*	*	9
Zhang YD	2016	*	*	*	*	**	*	*	*	9
Mihara Y	2017	*	*	*	*	*	*	*	*	8

Each "*" indicates one point for each item of the Newcastle -Ottawa Scale

**Table 3:** Bias risk in the randomized controlled trials (RCTs) as assessed by the Jadad scoring system

Studies	Year	Randomization	Blinding	Withdrawals and dropouts	Total
Matsuda K	2015	2	1	1	4
Niu JW	2016	1	0	1	2
Guo YC	2017	2	1	1	4
Zhou JM	2018	1	0	1	2

**Table 4:** Outcomes of interest reported in the 18 studies in patients receiving high or low ligation of the IMA

Study	Year	Surgical treatment of IMA (no. of patients)		Anastomotic leakage (no. of patients)		Overall morbidity (%)		Postoperative mortality (%)		Lymph nodes harvested (mean)		Overall recurrence rate (%)		5-year survival rate (%)	
		High ligation	Low ligation	High ligation	Low ligation	High ligation	Low ligation	High ligation	Low ligation	High ligation	Low ligation	High ligation	Low ligation	High ligation	Low ligation
Corder AP	1992	91	52	12	6										
Hall NR	1995	30	32	4	2										
Komen N	2011	16	17	1	1										
Sekimoto M	2011	27	45	0	1										
Rutegard M	2012	818	1101	81	108			1.6	1.5						
Hinoi T	2013	304	584	40	43	22.7	22.3			17.8	13.3				
Yamamoto M	2014	91	120	2	2							16.5	15.0	91.2	90.0
Bostrm P	2015	334	388	41	41			2.7	1.8						
Matsuda K	2015	51	49	8	5	35.3	20.4					1.96	2.0		
Huang J	2016	87	29	7	1										
Niu JW	2016	45	52	3	0										
Rutegard M	2016	5	18	1	2										
Yasuda K	2016	42	147	2	3	19.1	17.0					23.8	20.4	82.4	80.3
Zhang LY	2016	42	61	3	2					16.1	15.5				
Zhang YD	2016	84	132	2	0					14.9	14.3				
Guo YC	2017	29	28	3	1										
Mihara Y	2017	117	745	9	35	53.0	38.5			16.21	17.71				
Zhou JM	2018	52	52	2	0	5.8	9.6			16.9	24.9				

### Meta-analysis of the primary end point

**Fig. 2:**
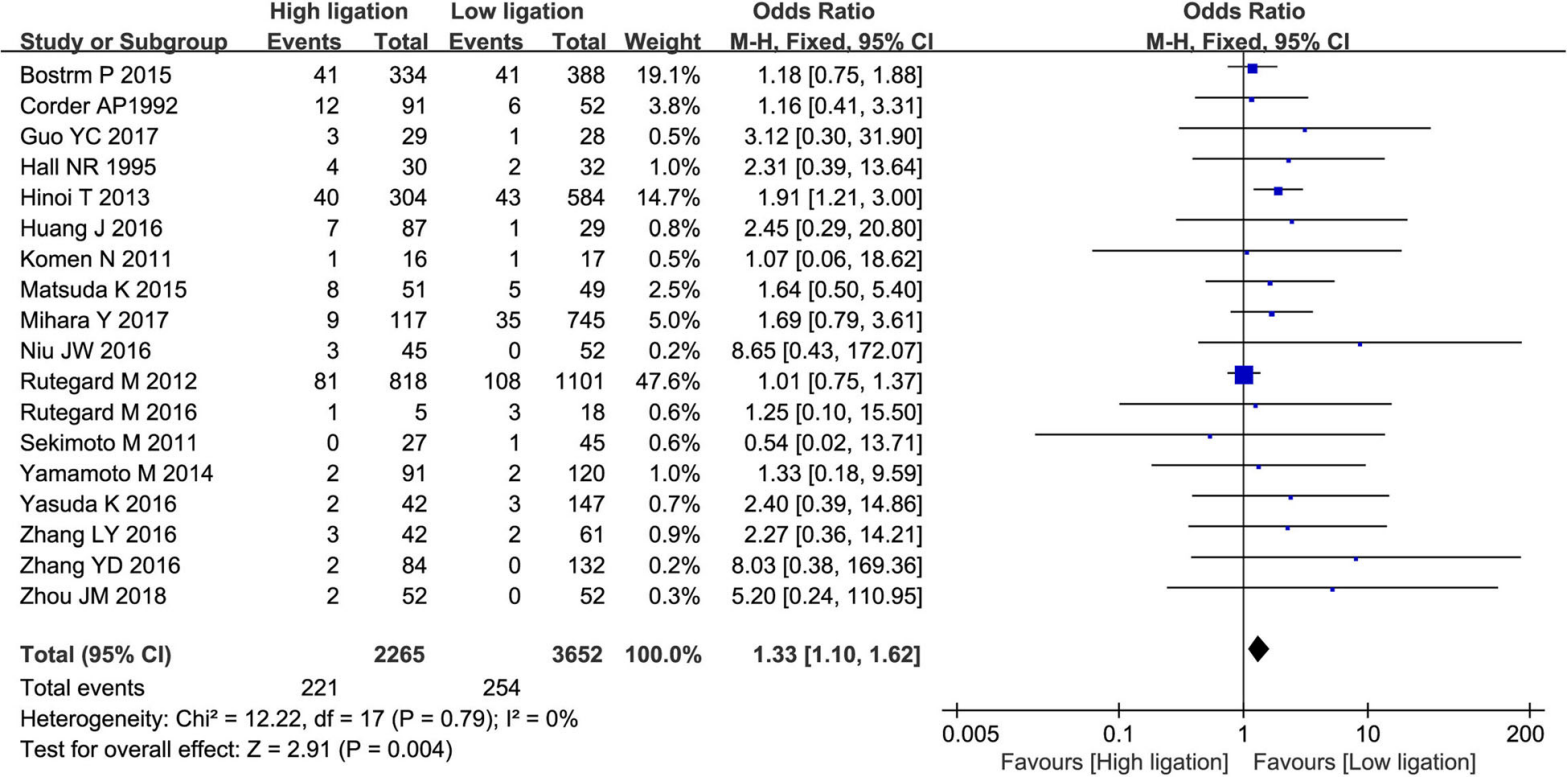
Forest plot for anastomotic leakage in both randomized and non-randomized studies

The pooled AL rate was 9.8% (221/2265) in the high ligation group and 7.0% (254/3652) in the low ligation group. The risk of AL was significantly greater in the high ligation group (OR, 1.33; 95% CI, 1.10–1.62; *P* = 0.004), without heterogeneity among the studies (*χ*^2^ = 11.22, *I*^2^ = 0%; Fig. 2).

**Fig. 3:**
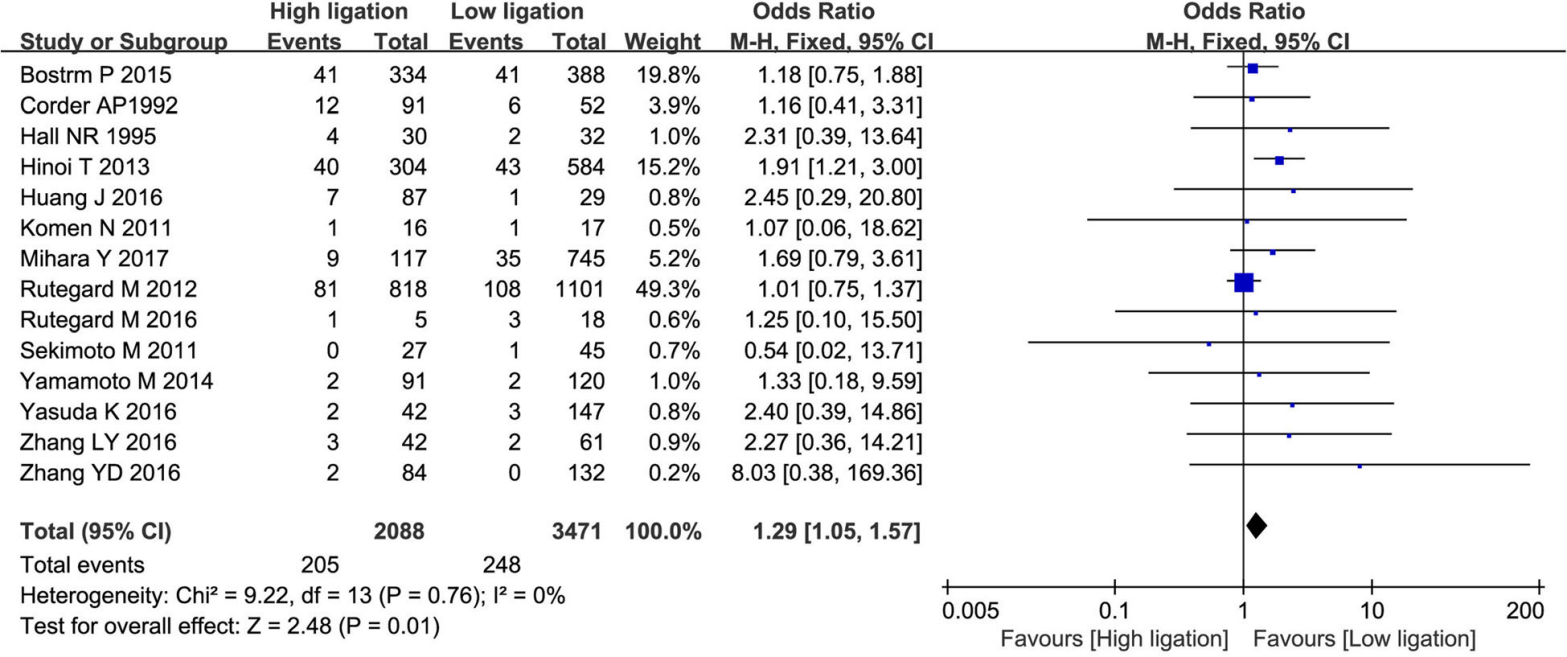
Forest plot for anastomotic leakage in non-randomized clinical studies only

In the 14 non-randomized clinical studies, the AL rate was 9.8% (205/2088) in the high ligation group and 7.1% (248/3471) in the low ligation group. The risk of AL was significantly higher in the high ligation group (OR = 1.29; 95% CI, 1.05–1.57; *P* = 0.01), without heterogeneity between the studies (*χ*^2^ = 9.22; *I*^2^ = 0%; Fig. 3).

**Fig. 4:**
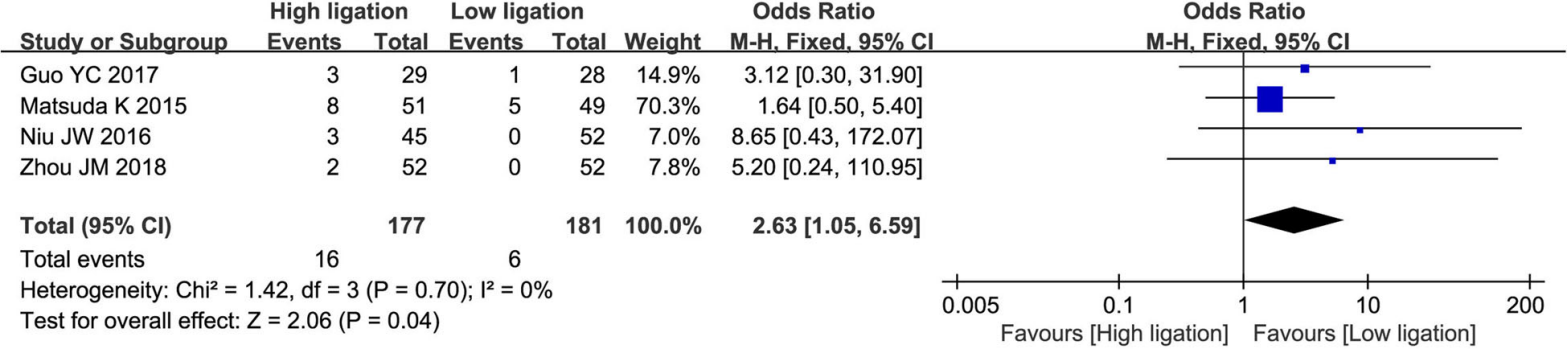
Forest plot for anastomotic leakage in randomized controlled trials only

In the four included RCTs, the risk of AL showed no heterogeneity between studies (*χ*^2^ = 1.42; *I*^2^ = 0%), and so a fixed effects model was adopted for the analysis. The AL rates were 9.0% (16/177) in the high ligation group and 3.3% (6/181) in the low ligation group. The risk of AL was significantly higher in the high ligation group (OR = 2.63; 95% CI, 1.05–6.59; *P* = 0.04; Fig. 4).

### Meta-analysis of the secondary end points

**Fig. 5:**
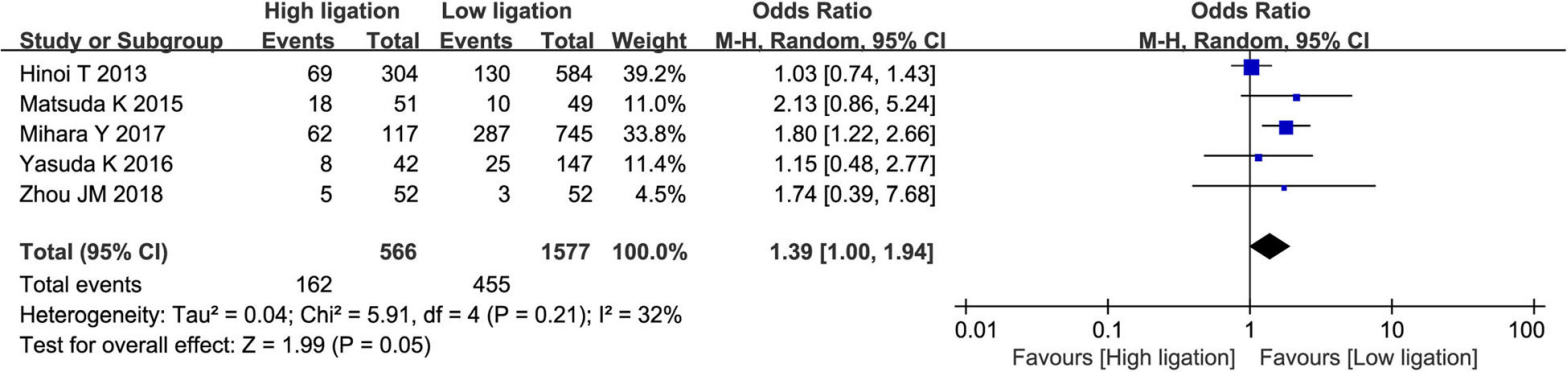
Forest plot for overall morbidity outcome

Overall morbidity was reported in only five studies [27, 30, 34, 38, 39]. There was substantial heterogeneity among the studies (*I*^2^ = 32%, *P* = 0.21), and so the random effects model was adopted. Pooled analysis revealed overall morbidity was significantly higher in high ligation patients (OR = 1.39; 95% CI, 1.05–1.68; *P* = 0.05; Fig. 5).

**Fig. 6:**
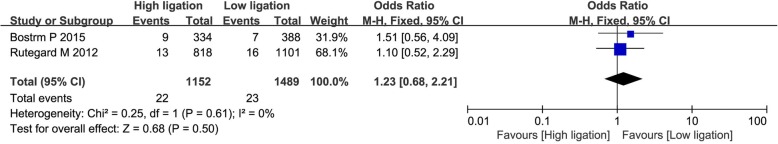
Forest plot for postoperative mortality outcome

Two studies [26, 29] reported postoperative mortality. There was no heterogeneity among the studies (*χ*^2^ = 0.25; *I*^2^ = 0%). The difference in postoperative mortality between the high ligation and low ligation groups was not statistically significant (OR = 1.23; 95% CI, 0.68–2.21; *P* = 0.50; Fig. 6).

**Fig. 7:**
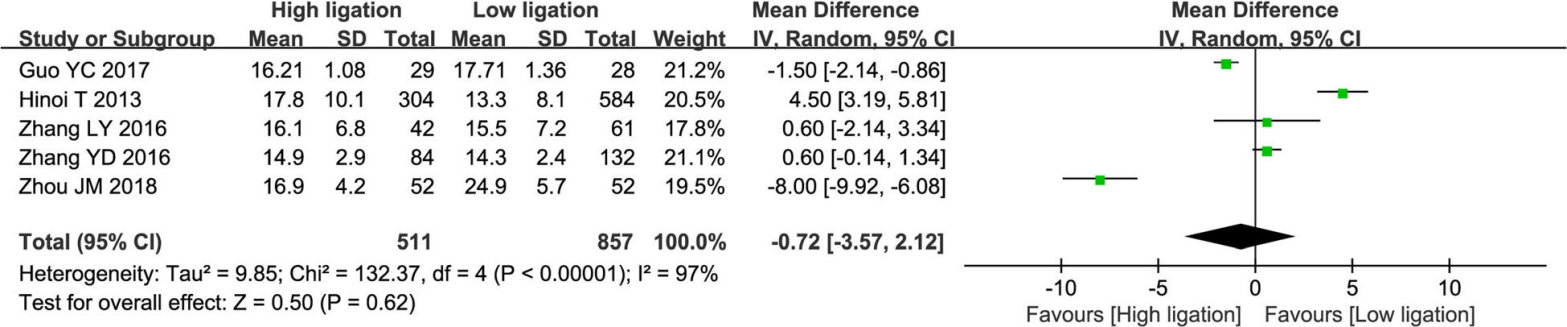
Forest plot for the number of harvested lymph nodes outcome

Five studies [27, 35–37, 39] compared the number of harvested lymph nodes. There was substantial heterogeneity among the studies (*I*^2^ = 97%), and so the random effects model was adopted. Pooled analysis showed no statistically significant difference between the high ligation group and the low ligation group (OR = − 0.72; 95% CI, − 3.57–2.12; *P* = 0.62; Fig. 7).

**Fig. 8:**
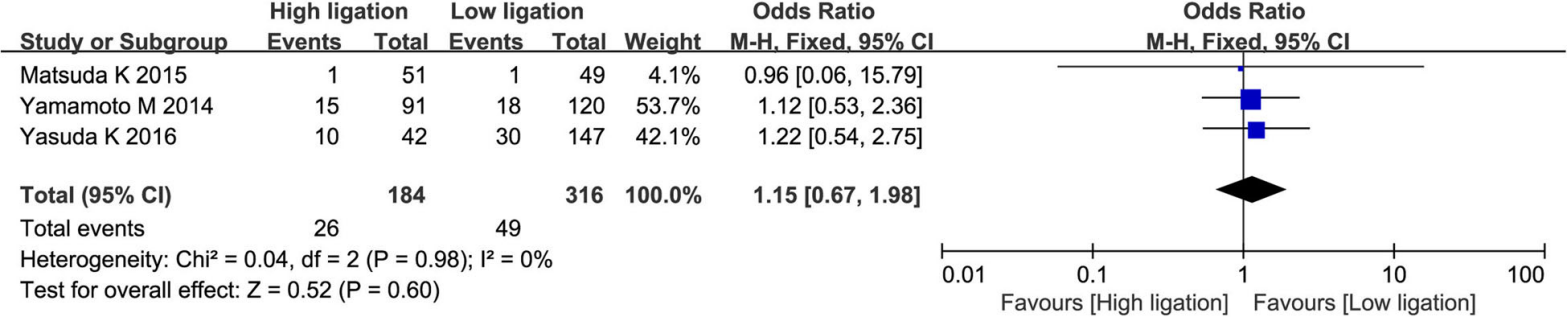
Forest plot for overall recurrence rate outcome

The overall recurrence rate was reported in three studies [28, 30, 34]. There was no heterogeneity among the studies (*χ*^2^ = 0.04; *I*^2^ = 0%), and so a fixed effects model was used. There was no significant difference in overall recurrence rate between the high ligation group and the low ligation group (OR = 1.15; 95% CI, 0.67–1.98; *P* = 0.60; Fig. 8).

**Fig. 9:**
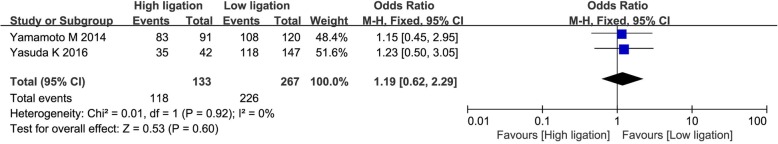
Forest plot for 5-year survival rate outcome

Two studies [28, 34] assessed the 5-year survival rate. There was no heterogeneity among the studies (*χ*^2^ = 0.01; *I*^2^ = 0%). Pooled analysis showed no significant difference in the 5-year survival rate between the high ligation group and low ligation group (OR = 1.19; 95% CI, 0.62–2.29; *P* = 0.60; Fig. 9).

### Sensitivity analysis

On NOS assessment, the 14 non-randomized studies showed high quality. However, the Jadad score was low for the studies of Niu et al. [32] and Zhou et al. [39] (Table 3). They did not describe the exact procedure of blinding, considering that the outcome analyzed in this study was AL. We excluded the studies with low Jadad score and recalculated the pooled OR for the primary end point (AL) in the remaining studies. However, in both non-randomized and randomized studies, the risk of AL remained higher in high ligation patients. Due to the low number of studies reporting the secondary end points, pooled sensitivity analysis for the secondary end points was not performed.

### Publication bias

**Fig. 10:**
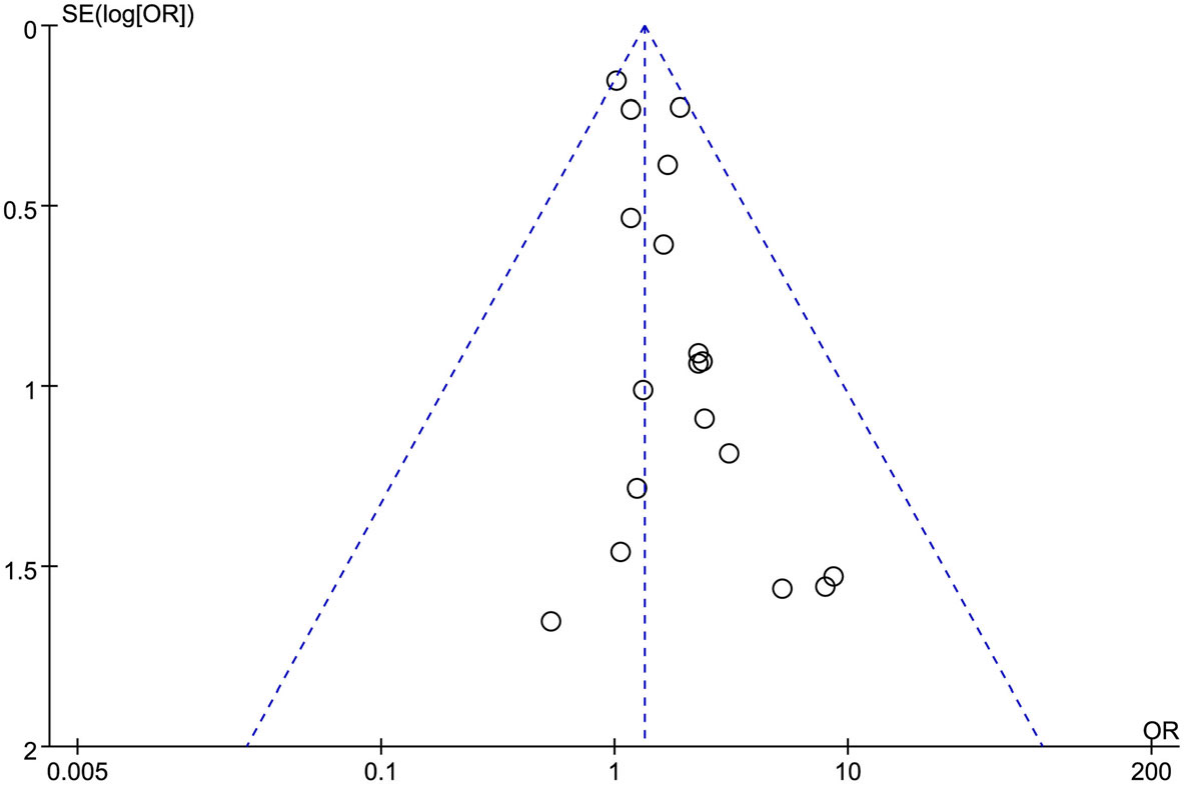
Funnel plot of anastomotic leakage

No significant publication bias was detected. As the funnel plot (Fig. 10) shows, the ORs of all the studies were within the pooled 95% confidence intervals. In addition, the studies were equally distributed on both sides of the vertical line.

## Discussion

In CRC surgery, AL is the most common serious postoperative complication and an important cause of mortality. It is still not certain whether high or low ligation of the IMA is better for reducing the risk of AL. The primary aim of this meta-analysis was to examine how the level of IMA ligation was associated with the risk of AL. Factors reported to be associated with AL [40] include anastomosis tension, hemorrhage, hypoalbuminemia, blood transfusion, operative field infection, longer operation time, and corticosteroid use.

Anastomotic ischemia and increased anastomosis tension are believed to be the major reasons for the development of AL [14, 41]. High ligation of the IMA is often indispensable to achieve a tension-free anastomosis during low anterior resection, and undoubtedly, blood supply is a crucial factor for the healing of colorectal anastomoses. Some surgeons believe that the marginal arteries are sufficient to guarantee adequate blood supply to the anastomosis in patients receiving high ligation of the IMA [42, 43], whereas others argue that LCA ligation would reduce blood supply to the proximal limb [11, 23, 24, 44]. In 2012, Bonnet et al. performed an anatomical study to examine how high and low ligation of the IMA affected colon length in CRC surgery and concluded that the remnant colon was 10 cm longer when high ligation was performed [45]. However, in another anatomical study, Buunen et al. found that low ligation provided sufficient remnant colon length to guarantee a tension-free anastomosis in 80% of their specimens [46]. Komen et al. [24] used laser Doppler flowmetry to compare colonic perfusion after high or low ligation of the IMA in rectal surgery and reported that the blood flow ratio was higher when patients received low ligation, suggesting that the anastomosis might benefit from low ligation of the IMA. A prospective cohort study conducted by Hinoi et al. [27] in 2013 showed that LCA preservation (i.e., low ligation of the IMA) in laparoscopic anterior resection for middle and low rectal cancer was associated with a lower AL rate.

Some recently published meta-analyses [5, 8–10] that compared outcomes after CRC surgery found no statistically significant differences between high ligation patients and low ligation patients in 5-year survival rate, lymph node yield, postoperative morbidity, postoperative mortality, overall recurrence rate, incidence of bowel obstruction, intraoperative blood loss, operation time, surgical site infection, postoperative bleeding, and so on. However, the AL rate differed between the two sets of patients. Fan et al. [9] analyzed the division branches of the IMA in sigmoid colon and rectal cancer surgery and concluded that low ligation of the IMA may be less likely to cause AL. Cirocchi et al. and Yang et al. [5, 8] analyzed high tie and low tie of the IMA in CRC surgery and showed that there was no significant difference between the two approaches. It should be noted that Cirocchi et al. and Yang et al. had a fewer number of studies with data on AL than did Fan et al., and so their results may be less reliable. We included both randomized and non-randomized studies in our meta-analysis and found that low ligation of the IMA in sigmoid colon and rectal cancer surgery could significantly reduce the AL rate (*P* = 0.004). Analysis of data from non-randomized studies and RCTs separately also showed that high ligation was associated with higher risk of AL (*P* = 0.01 and *P* = 0.04, respectively). Unfortunately, the number of RCTs in our meta-analysis was small and a lack of the data on the incidence of anastomotic leakage in the RCTs. Furthermore, there were obvious limitations in the data available. Most of the data were from non-randomized clinical studies performed over a decade ago, and it is likely that there were many differences in the management of sigmoid colon and rectal cancer over this period.

We found no significant differences between high ligation and low ligation patients in postoperative mortality, number of harvested lymph nodes, overall recurrence rate, and 5-year survival rate. This is consistent with previous meta-analyses. We found higher incidence of overall morbidity in high ligation patients; higher incidence of AL is probably the major reason. Although it is to be expected that higher incidence of AL will also increase postoperative mortality, we did not find any significant increase in this outcome in the high ligation group. This may have been because of the small number of studies and incomplete reporting of this outcome. Several authors have reported that low ligation of the IMA in CRC surgery is associated with decrease in the number of harvested lymph nodes [47, 48]. However, we did not find a statistically significant difference between the two groups in the number of lymph nodes harvested. Furthermore, in both groups, the number of lymph nodes harvested was > 12, which meets the recommendation in the National Comprehensive Cancer Network guidelines [49]. Some authors have reported that AL after CRC surgery is associated with cancer recurrence and poor prognosis [50]. We did not find any association between level of ligation and survival or recurrence after CRC, but this too may have been because of the small number of included studies and the lack of relevant data in the four RCTs in this meta-analysis.

## Conclusions

Low IMA ligation during curative resection of sigmoid colon and rectal cancer appears to be associated with relatively higher risk of AL and overall morbidity. However, postoperative mortality, number of harvested lymph nodes, overall recurrence rate, and 5-year survival rate do not seem to be affected by the level of ligation. Large well-designed multicenter randomized clinical trials are necessary to confirm these findings and throw more light on this topic.

## Abbreviations

AL: Anastomotic leakage
CI: Confidence interval
CRC: Colorectal cancer
IMA: Inferior mesenteric artery
LCA: Left colic artery
NOS: Newcastle–Ottawa Scale
OR: Odds ratio
RCT: Randomized controlled trial
WMD: Weighted mean difference
